# Holiday Reindeer Trivia for Physicians Who Like Hair and Nails

**DOI:** 10.1159/000521326

**Published:** 2022-01-10

**Authors:** Julia Wells, Kylie Watson, C. Ralph Daniel, Robert T. Brodell, Vinayak K. Nahar

**Affiliations:** ^a^Center for Animal and Human Health in Appalachia, College of Veterinary Medicine, Lincoln Memorial University, Harrogate, Tennessee, USA; ^b^Department of Dermatology, University of Mississippi Medical Center, Jackson, Mississippi, USA; ^c^Associate Professor of Dermatology and Preventive Medicine, University of Mississippi Medical Center, Jackson, Mississippi, USA

Reindeer horns and hoofs are made of keratin and are similar in their production to hair and nails. The word reindeer comes from the Old Norse word hreinin which means “horned animal.” Keratin is derived from the Latin cornu and predated by the Greek word Keras (κερας) which means horn. Keras was also used as a drinking container by ancient Greeks and later Vikings. Reindeer hooves enable these animals to walk long distances in harsh weather conditions. Anatomically, their hooves follow a similar pattern to other ungulates such as cattle, sheep, and deer. They have 4 digits, each comprising 3 phalanges, a sesamoid bone, and hoof capsules encircling the middle and distal phalanges of digits 2 and 3 [1]. Reindeer bear their weight predominantly using 2 digits, with the incorporation of their dewclaws to aid in navigating the snowy terrain encountered in the North Pole. When walking, reindeer's foot pads and hoof capsules expand and flatten to absorb the weight cushioning the blow of each step [2]. The outer hoof capsules in reindeer function to protect and contain structures within the hoof, and it is comparable to the nail plate in human fingernails and toenails. Beneath the hoof capsule, reindeer contain sensitive and insensitive laminae structures with capillaries to provide blood flow to the hoof. These structures are comparable to the nail bed in human nails, which consists of an outer epidermis and an inner, vascular dermis (Fig. 1).

Reindeer antlers are osseous projections that grow out of an anchor-like pedicle in the frontal bone. Reindeer are not born with antlers − they must undergo hormonal changes that cause their development later in life. While developing, reindeer antlers are covered with a velvet-like material that is similar to human hair. This velvet provides nutrients to the antlers as they grow. When they are fully grown, reindeer antlers will shed their velvet and instead have a smooth surface and numerous points [3]. At this point, reindeer antlers do not resemble human hair at all, as they are made entirely of bone, compared to the keratin comprising human hair. One important distinction of reindeer and other antler-growing animals is that both male and female reindeer grow antlers! This can make it difficult to tell male and female reindeer apart, even while standing on the ground watching the reindeer fly above.

Although seemingly unrelated, hair follicles and antlers have some striking similarities, including both structural components and the developmental process. Both hair follicles and antlers can be divided into a permanent and a temporary/cyclic structure. These mammalian organs rely on stem cells for regeneration, which are found in the permanent part and a growth center, which is found in the temporary part of the antler and hair follicle [4]. Hormonal influence is very important in regulating the development of both hair follicles and antlers.

As we enter the holiday season, certain information may prove valuable to the audience of physicians with a special interest in compact keratin of the hair and nails. Below are 4 fun facts about our reindeer friends to take with you to the holiday gatherings this season.

1. Reindeer are domesticated caribou sharing the same genus and species (*Rangifer tarandus*). They are not capable of living in the wild without the support of humans, perhaps including Santa Claus.
2. There is documentary evidence of Santa's reindeer having horns in the holiday season. All of Santa's reindeer, therefore, were female and “with child” despite their names that might suggest otherwise. Male deer lose their antlers in the winter. Female reindeer also lose their antlers in the winter except when they are pregnant. The evolutionary significance of this might be to protect their brood that are soon to be born.
3. Reindeer do click as suggested in the song, *Up on the House Top,* by Benjamin Hanby in 1864. You will remember the lyric, “Up on the housetop, click, click, click; Down through the chimney with old Saint Nick.” The clicking is NOT related to the clatter of hard keratin against a tin roof. Rather, reindeer have a ligament attached to their hoofs that slips when they walk down steep hills causing a clicking sound. Evolutionarily this sound might help keep the herd together as they walk down steep mountains in snow when visibility is obstructed.
4. Reindeer/caribou hooves are also adaptable. In the summer, the foot pads are soft to provide extra grip on wet surfaces. In the winter, the foot pads tighten exposing the rim of their hoof for extra traction in snow and ice.

This brief review of horns and hoofs is designed to highlight the phylogeny of hair and nails while taking a light-hearted look at Santa's mammalian helpers. While nails are largely vestigial organs, they played a significant role in human ancestors and other mammals. Even today, human nails have important functions, including protection against an aggressor, chemical analysis for toxins and other substances can lead to a diagnosis, aid in fine touch and grasp, help dermatologists diagnose local and systemic diseases, protect the distal digit, and cosmetic/decorative purposes. It is the authors' hope that everyone has a happy and healthy holiday season.

## Conflict of Interest Statement

Robert T. Brodell has participated in multicenter clinical trials with Corevitas (formerly Corrona) Psoriasis Registry and Novartis. He has received grant from Pfizer. He is also an associate editor of the *Journal of the American Academy of Dermatology*, Faculty advisor for the *American Medical Student Research Journal*, and editor-in-chief of *Practice Update: Dermatology* and serves as Staff Dermatologist at the GV (Sonny) MONTGOMERY VA HOSPITAL in Jackson, MS. Daniel III C. Ralph is board of directors of Council for Nail Disorders, European Nail Society, and St. Dominic Health Services Foundation. He is also a Clinical Professor of Dermatology at the University of Alabama at Birmingham. He is also American Dermatological Association Chairman of the Endowment Committee. He serves on the editorial board of the *Skin Appendages Disorders* and on the advisory board of *Ortho Pharmaceutical*. He is also co-editor of a book Scher and Daniel's Nails. 4th edition, Springer, Philadelphia, 2018. He is also stakeholder of Medimetriks. Vinayak K. Nahar has received grants from Banfield Pet Hospital and Pfizer. Julia Wells and Kylie Watson have no conflicts of interest.

## Funding Sources

This study did not receive any funding.

## Author Contributions

R.T.B. and V.K.N. contributed to conception and design; all authors drafted the article or revised the manuscript critically for important intellectual content; all authors gave final approval of the version of the article to be published; all authors agree to be accountable for all aspects of the work in ensuring that questions related to the accuracy or integrity of any part of the work are appropriately investigated and resolved; and all authors have read and approved the manuscript.

## Figures and Tables

**Fig. 1:**
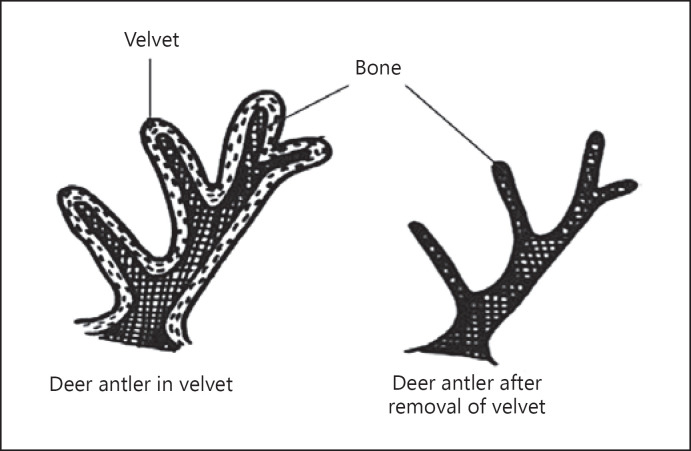
During the antler-growing process, it is covered in velvet, a furry membrane that provides blood and nutrients. At the end of the growing cycle, the antlers will harden and the velvet will shed (https://commons.wikimedia.org/wiki/File:Anatomy_and_physiology_of_animals_Deer_antler.jpg).
